# Respiratory Viruses Associated Hospitalization among Children Aged <5 Years in Bangladesh: 2010-2014

**DOI:** 10.1371/journal.pone.0147982

**Published:** 2016-02-03

**Authors:** Nusrat Homaira, Stephen P. Luby, Kamal Hossain, Kariul Islam, Makhdum Ahmed, Mustafizur Rahman, Ziaur Rahman, Repon C. Paul, Mejbah Uddin Bhuiyan, W. Abdullah Brooks, Badrul Munir Sohel, Kajal Chandra Banik, Marc-Alain Widdowson, Melisa Willby, Mahmudur Rahman, Joseph Bresee, Katharine-Sturm Ramirez, Eduardo Azziz-Baumgartner

**Affiliations:** 1 International Centre for Diarrhoeal Disease Research, Bangladesh (icddr,b), Dhaka, Bangladesh; 2 Centers for Disease Control and Prevention (CDC), Atlanta, Georgia, United States of America; 3 Bloomberg School of Public, Department of International Health, Johns Hopkins University, Baltimore, Maryland, United States of America; 4 Institute of Epidemiology Disease Control and Research, (IEDCR), Dhaka, Bangladesh; Université Paris Descartes ; AP-HP, Groupe Hospitalier Cochin-Saint-Vincent-de-Paul, FRANCE

## Abstract

**Background:**

We combined hospital-based surveillance and health utilization survey data to estimate the incidence of respiratory viral infections associated hospitalization among children aged < 5 years in Bangladesh.

**Methods:**

Surveillance physicians collected respiratory specimens from children aged <5 years hospitalized with respiratory illness and residing in the primary hospital catchment areas. We tested respiratory specimens for respiratory syncytial virus, parainfluenza viruses, human metapneumovirus, influenza, adenovirus and rhinoviruses using rRT-PCR. During 2013, we conducted a health utilization survey in the primary catchment areas of the hospitals to determine the proportion of all hospitalizations for respiratory illness among children aged <5 years at the surveillance hospitals during the preceding 12 months. We estimated the respiratory virus-specific incidence of hospitalization by dividing the estimated number of hospitalized children with a laboratory confirmed infection with a respiratory virus by the population aged <5 years of the catchment areas and adjusted for the proportion of children who were hospitalized at the surveillance hospitals.

**Results:**

We estimated that the annual incidence per 1000 children (95% CI) of all cause associated respiratory hospitalization was 11.5 (10–12). The incidences per 1000 children (95% CI) per year for respiratory syncytial virus, parainfluenza, adenovirus, human metapneumovirus and influenza infections were 3(2–3), 0.5(0.4–0.8), 0.4 (0.3–0.6), 0.4 (0.3–0.6), and 0.4 (0.3–0.6) respectively. The incidences per 1000 children (95%CI) of rhinovirus-associated infections among hospitalized children were 5 (3–7), 2 (1–3), 1 (0.6–2), and 3 (2–4) in 2010, 2011, 2012 and 2013, respectively.

**Conclusion:**

Our data suggest that respiratory viruses are associated with a substantial burden of hospitalization in children aged <5 years in Bangladesh.

## Introduction

Acute lower respiratory infections (ALRIs) are one of the leading causes of childhood morbidity and mortality globally [1]. They are also the leading causes of childhood death in Bangladesh [2–4]. Respiratory viruses are increasingly recognized as major contributors to acute respiratory infections (ARI) in children and associated with almost 60% of ALRIs [5]. The primary viral etiological agents for childhood ALRI are respiratory syncytial virus (RSV), influenza A and B viruses, rhinovirus, adenovirus, parainfluenza viruses (PIV) and human metapneumovirus (HMPV) [6–12].

Data from Bangladesh suggest that incidence of respiratory virus associated pneumonia in children aged <2 years was 40/100 child years with the incidence being highest for RSV [13, 14]. The rate per1000 child-years for RSV associated hospitalization ranges between 4 and 10 and for influenza between 1 and 2 in other low and middle income countries [15–17]. Although there is growing body of literature from low income settings, still information remains inadequate about the contribution of respiratory viruses to severe childhood respiratory illness. Data about the incidence of hospitalization may help health officials explore the potential value of targeting influenza vaccines to specific subpopulations presumed to be at higher risk of severe influenza illness in countries like Bangladesh where there is still no uniform policy guideline for influenza vaccination. In addition these data may help prioritize the development of vaccines for other respiratory viruses including RSV and parainfluenza viruses. Data on incidence of respiratory virus associated pediatric hospitalization are also necessary to estimate the total direct and indirect cost of hospitalization and the burden on the health care delivery system associated with childhood respiratory illness. We combined health utilization survey and hospital based surveillance data to estimate the incidence of respiratory virus infection among children aged <5years hospitalized with respiratory illness in Bangladesh.

## Methodology

### Study Design

The study was a combination of prospective hospital based surveillance and community based health care utilization survey.

### Study setting

#### Hospital-based surveillance

In 2010, the Institute of Epidemiology, Disease Control and Research (IEDCR) of the Government of Bangladesh and the icddr,b (International Centre for Diarrhoeal Disease Research, Bangladesh) initiated childhood respiratory illness surveillance in one private and three government tertiary level hospitals (Jahurul Islam Medical College Hospital, Kishoreganj, Comilla Medical College Hospital, Comilla, Shaheed Ziaur Rahman Medical College Hospital, Bogra and Sher-e-Bangla Medical College Hospital, Barisal) of Bangladesh. These four hospitals were selected from 12 icddr,b surveillance hospitals, based on geographical diversity and high turnover of patients within the influenza surveillance project. Investigators identified the primary catchment areas of the hospitals as the areas where the majority of the patients resided (an arbitrary cut-off of sub-districts where >60% of the patients hospitalized with respiratory symptoms resided) by reviewing the hospital log books.

#### Health care utilization survey

In order to estimate the proportion of children in the catchment area that sought care at the surveillance hospitals, we surveyed randomly selected households from each of the primary catchment areas of the four surveillance hospitals. The selections of households were computer generated at random and distributed by sub district with a probability proportional to the population of the sub-district. We used ArcGIS to randomly generate GPS (global positioning system) points that corresponded to the number of households to be surveyed from each sub-district. We plotted the randomly generated coordinates on Google^™^ map to locate each area. Our field team used handheld GPS devices to reach the coordinates. Once the field team reached the location of the GPS coordinates, the household in the immediate vicinity was selected as the first household for enrolment. Our field team then skipped the next two closest households on the right of the first enrolled household and selected the third household for enrolment. If a household did not consent to participate or was not present at the time of the survey, we skipped the next two closest households and approached the third household for the survey.

#### Study participants

Beginning January 2010, once every two weeks, surveillance physicians in each surveillance hospital enrolled all children aged<5 years residing in the primary catchment area and hospitalized with any two of the following symptoms: reported/measured fever, cough or difficulty breathing at the time of admission. In January 2011, we increased the number of surveillance days from once every two weeks to once every week.

Beginning January 2012, to better understand the causal association between infection with a respiratory virus and the presence of respiratory symptoms, we enrolled all children aged <5 years residing in the primary catchment areas, who were hospitalized on the sampling day (control children) but did not have any respiratory symptoms (including cough, difficulty breathing, runny nose or sore throat) in the 7 to 14 days preceding hospitalization or on the day of admission.

Between January 1 and March 31 2013, field researchers identified households selected for the health care utilization survey with children aged <5 years who had been hospitalized with any two of respiratory symptoms including reported/measured fever, cough and difficulty breathing within the preceding 12 months at any of the four surveillance hospitals versus other area health facilities.

#### Data sources

The surveillance physicians followed each enrolled child throughout hospitalization and collected clinical information using a structured questionnaire. We assumed that influenza and RSV-associated deaths would most frequently occur within two weeks of illness [18]. Therefore, we called parents/guardians of each child after 14 days of enrollment in the surveillance to inquire if the child had fully recovered, developed any further respiratory symptoms, required re-hospitalization or had died. If a child's guardian was unavailable by telephone, the field assistant made a follow-up visit to his/her home.

As part of the health care utilization survey, field assistants collected information regarding history of respiratory hospitalizations of children aged < 5 in the household, age and sex of the child, symptoms of the respiratory illness, where the child was hospitalized, and the reason for choosing the particular facility.

#### Biases

To have a good representation of the country, the surveillance hospitals were chosen from four geographically diverse areas of the country. One of the four hospitals was private which adjusted for the variation in care between private and public facilities. All the four hospitals were tertiary level teaching hospitals thus the level of care provided in each of the surveillance hospitals was similar. On surveillance days, all children who met our case definition were approached for participation in the surveillance to minimize selection bias. Sampling days were spread throughout all days of the week except for the one day the hospital administration was closed (Friday for the three government hospitals and Tuesday for the private hospital).

#### Sample size

As the study was part of a surveillance we did not do any specific sample size calculation for the surveillance. We aimed to enroll all eligible children on the sampling day. We accounted for the sampling frame in the analyses of the data.

The health care utilization survey was conducted as part of a larger health care utilization survey. For the health care utilization survey, our outcome of interest was the proportion of people with respiratory symptoms who visited the 12 surveillance hospitals across the country where we had our ongoing influenza surveillance. We assumed that 0.6% of the people living in the hospital catchments areas had acute respiratory symptoms and sought care at the surveillance hospital. For a precision of 0.3% and 80% power, we needed a sample of 2,546 persons across all age groups. We included a design effect of 2.5 to account for clustering effect and yielded a sample size of 6,364 persons. We used four persons as the mean household size in Bangladesh [29], thus calculated we needed to survey 1,591 households. Since we accounted for cluster design effect of 2.5 our effective sample size was 3,978 households across all catchment areas.

#### Sample collection and laboratory analyses

Surveillance physicians collected nasal and throat swabs from each enrolled child (symptomatic and control). Immediately after collection, field assistants stored the samples in nitrogen dewars and transported to icddr,b within 15 days of collection. At the icddr,b virology laboratory, specimens were tested for RSV, PIV types 1–3, HMPV, influenza viruses (influenza A and B), rhinovirus and adenovirus by real-time reverse transcription polymerase chain reaction (rRT-PCR) using primers and probes supplied by the U.S. Centers for Disease Control and Prevention (CDC) [19, 20]. However, due to logistical constraints we could not test the samples for rhinovirus in 2014.

#### Data analyses

We used the census data from 2011[21] to estimate the number of children aged <5 years living in the primary catchments areas of the hospitals. We assumed an annual population growth rate of 1.3% [21] to estimate the population in the catchment areas during 2010, 2012, 2013 and 2014.

Our surveillance was active one day every two weeks in 2010 and one day every week in 2011–2014. We estimated the number of respiratory hospitalizations during the entire month by multiplying the number of children enrolled monthly in surveillance on the sampling days by the proportion of days when surveillance was active during that month (30.4/2 in 2010 and 30.4/4 in 2011–2014). We divided the estimated annual number of hospitalized respiratory cases by the census population aged <5 years to estimate the annual incidence. We then adjusted the result for the proportion of children who lived in the catchment area and were hospitalized at the sentinel hospital out of all children who lived in the catchment area and were hospitalized at any hospital for respiratory illness. We calculated the 95% confidence interval of the annual incidences using the delta method [22].

We estimated the monthly number of laboratory-confirmed respiratory virus specific infections among hospitalized children by multiplying the monthly positivity for each respiratory virus by the estimated number of children hospitalized with respiratory illness in the same month. We estimated the annual rates of virus specific infection among hospitalized children by dividing the estimated annual number of hospitalizations with RSV, rhinovirus, adenovirus, PIVs, HMPV or influenza-associated respiratory infection by the year-specific census population aged <5 years and adjusted the result for the proportion of the catchment population aged <5 years hospitalized at the surveillance hospitals with respiratory symptoms. To account for variation in hospitalization on week days compared to on weekly holidays, we accounted for the difference in the estimated number of hospitalizations on week days compared to weekly holidays (the mean number of respiratory hospitalization on week days was 1.3 compared to one on weekly holidays) in our incidence calculation.

To calculate confidence intervals, we randomly selected a sample of size n_i_ from the number of children sampled during each month (n_i_ = number of samples obtained in the i-th month, i = 1, 2, 3 … 12) with replacement from the observations obtained from i-th month. Then we combined the random samples from the 12 months of a year to obtain a nonparametric bootstrap sample and estimated the parameters of interest (e.g. number of children hospitalized with respiratory illness and the number of children hospitalized with RSV-associated respiratory illness on a specific month). We repeated the three steps 10,000 times and took the 2.5^th^ and 97.5^th^ percentiles from the estimated values of the parameter to calculate the 95% CI for the parameter [23]. In calculating the 95% CI we assumed that the number of samples collected on the specific sampling day each week would be similar if we collected samples every day of the week and could not account for the potential daily variability. In addition, our equation for analyses was stratified by month assuming that the four days selected for each month were representative of all the other days in a month. We also assumed the proportion of samples testing positive was sufficiently captured by the four monthly days of sampling and were representative for that month when indeed, there may have been greater variability unaccounted for within the month.

We also collected respiratory specimens from asymptomatic children beginning in 2012, we used descriptive statistics to present the distribution of respiratory viral pathogens in these control children.

#### Protection of human subjects

The surveillance physician obtained written informed consent from the parents or guardians of the children enrolled in the surveillance. The Institutional Review Board (IRB) at icddr,b (International Centre for Diarrhoeal Disease Research, Bangladesh) approved the research protocol. CDC reviewed and approved reliance on icddr,b’s IRB approval.

## Results

### Hospital based surveillance

#### Clinical findings and laboratory diagnosis

**Fig 1:**
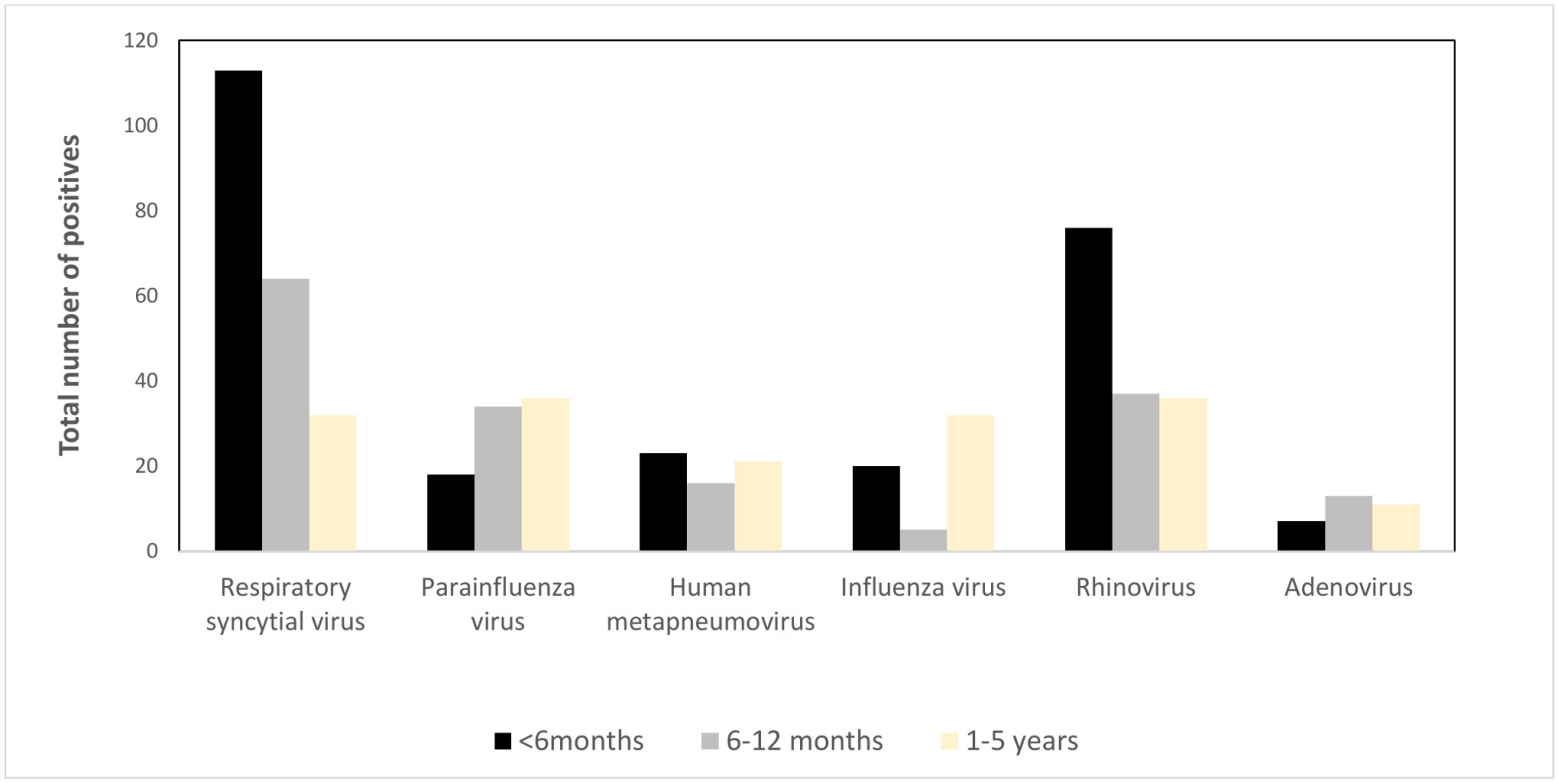
Cumulative age-specific viral etiology of hospitalized children with respiratory illness aged <5 years enrolled in the four surveillance hospitals, Bangladesh, 2010–2014.

Surveillance physicians enrolled 155 children hospitalized with respiratory illness in 2010, 209 in 2011,153 in 2012, 128 in 2013, and 184 in 2014 (Table 1). Of the 829 children, 580 (70%) were male. The median age of the children was 5 months (IQR [interquartile range] 2–10 months). The median age for children enrolled in 2010, 2011, 2012,was 5 months, for 2014 was 4 months and only for 2013 was 7 months. Children were hospitalized a median of 3 days (IQR 2–4 days) after symptoms onset and remained hospitalized for a median of 2 days (IQR 0–4 days). The main clinical symptoms and signs of case-patients as reported by the attending physicians were cough (99%), difficulty breathing (87%), measured fever (84%), runny nose (65%), chest indrawing (65%), crepitation (48%), ronchi (46%) and wheezing (35%). Family members of 25 children (3%) reported that their child had previous history of asthma/recurrent wheeze. Among the 829 children enrolled, 486 (55%) were laboratory-confirmed for infection with one of the seven respiratory viruses (Table 1). In addition 15% of the enrolled children with respiratory symptoms were also positive for more than one virus and almost 30% (27 of 95) of the respiratory specimens positive for viral co-infection were parainfluenza virus with adenovirus or rhinovirus and human metapneumovirus with adenovirus or rhinovirus. Hospitalized children aged less than six months most commonly had laboratory-confirmed RSV (129 of 459, 28%) (Fig 1).

**Table 1:** The frequency of respiratory viral infections in children aged <5 years hospitalized with and without respiratory symptoms in four surveillance hospitals, Bangladesh 2010–2014.

Respiratory viral pathogens	Frequency (%)									
	Hospitalized children with respiratory symptoms						Hospitalized children without respiratory symptoms			
	2010	2011	2012	2013	2014	2010–2014	2012	2013	2014	2012–2014
	n = 155	n = 209	n = 153	n = 128	N = 184	n = 829	n = 246	n = 174	n = 119	n = 540
Any positive	143 (92)	145 (69)	106 (69)	106 (83)	121 (66)	**621 (75)**	117 (47.5)	94 (53)	22 (18)	**233**
Respiratory syncytial virus	69 (44.5)	39 (19)	35 (23)	8 (6)	46 (25)	**197 (24)**	9 (4)	0 (0)	3 (2.5)	**12 (2)**
Rhinovirus	40 (31)	37 (18)	21 (14)	51 (40)	Not tested	**Not applicable**	41(17)	47 (27)	Not tested	**Not applicable**
Adenovirus	9 (6)	8 (3)	4 (3)	5 (4)	5 (3)	**31 (4)**	38 (15)	19 (11)	7 (6)	**64 (12)**
Parainfluenza viruses1-3	6 (4)	16 (8)	8 (5)	6 (5)	9 (5)	**45 (5)**	4 (2)	4 (2)	5 (4)	**13 (2)**
Human metapneumovirus	4 (2.5)	14 (7)	3 (2)	7 (5)	6 (3)	**34 (4)**	0 (0)	3(2)	0 (0)	**3 (0.5)**
Influenza viruses	4 (2.5)	6 (3)	3 (2)	4 (3)	8 (4)	**25 (3)**	3 (1)	1 (0.5)	1 (1)	**5 (1)**
Viral co-infections	11 (7)	25 (12)	32 (21)	25(19.5)	31 (17)	**95 (15)**	22 (9)	19 (11)	6 (5)	**47 (9)**
Negative	12 (8)	64 (31)	47 (53.5)	22 (17)	63 (34)	**208 (25)**	129 (52)	81 (46.5)	97 (81.5)	**307 (57)**

We obtained information about the survival status of 99% (821 of 829) of the children after discharge beyond two weeks of symptoms onset. From January 2010 through December 2014, we identified 13 deaths (case fatality proportion 1.6% 95% CI 0.8–2.7%). Of the 13 death cases 6 (46%) were male and the median age was 3 months (IQR 2–5 months). One of the 197 children with laboratory-confirmed RSV (in hospital fatality of 0.7%, 95% CI 0.01–2.8%), two of the 149 with rhinoviruses (in hospital fatality of 1.3%, 95% CI 0.16–4.8%) and two of the 34 with HMPV (in hospital fatality of 6%, 95% CI 0.7–21%) died within 14 days of enrollment into the surveillance. We did not identify any deaths among the 25 influenza, 45 parainfluenza or 31 adenovirus infected children.

#### Profile of the asymptomatic control children

**Fig 2:**
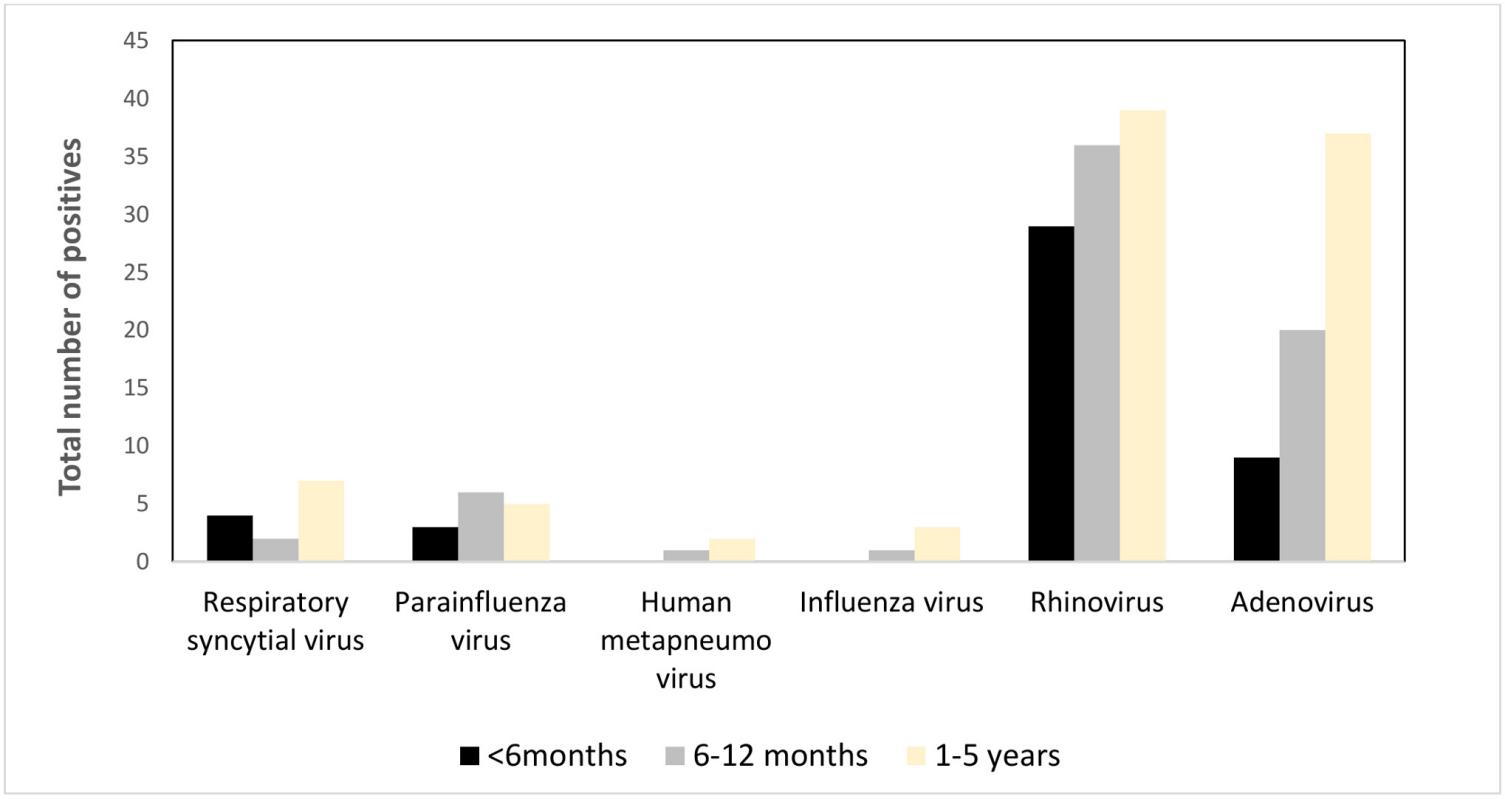
Cumulative age-specific viral etiology of hospitalized children without respiratory illness aged <5 years enrolled in the four surveillance hospitals, Bangladesh, 2010–2014.

We enrolled 246 children in 2012, 175 in 2013 and 119 children in 2014 children who did not have any respiratory symptoms in the preceding 7–14 days before admission (Table 1). Out of these 540 children, 37.5% of them were positive for one of the respiratory viruses and their median age was 12 months (IQR 7–18 months). Rhinovirus and adenovirus were frequently identified among asymptomatic children across all age groups (Fig 2).

### Annual distribution of viral activity

**Fig 3:**
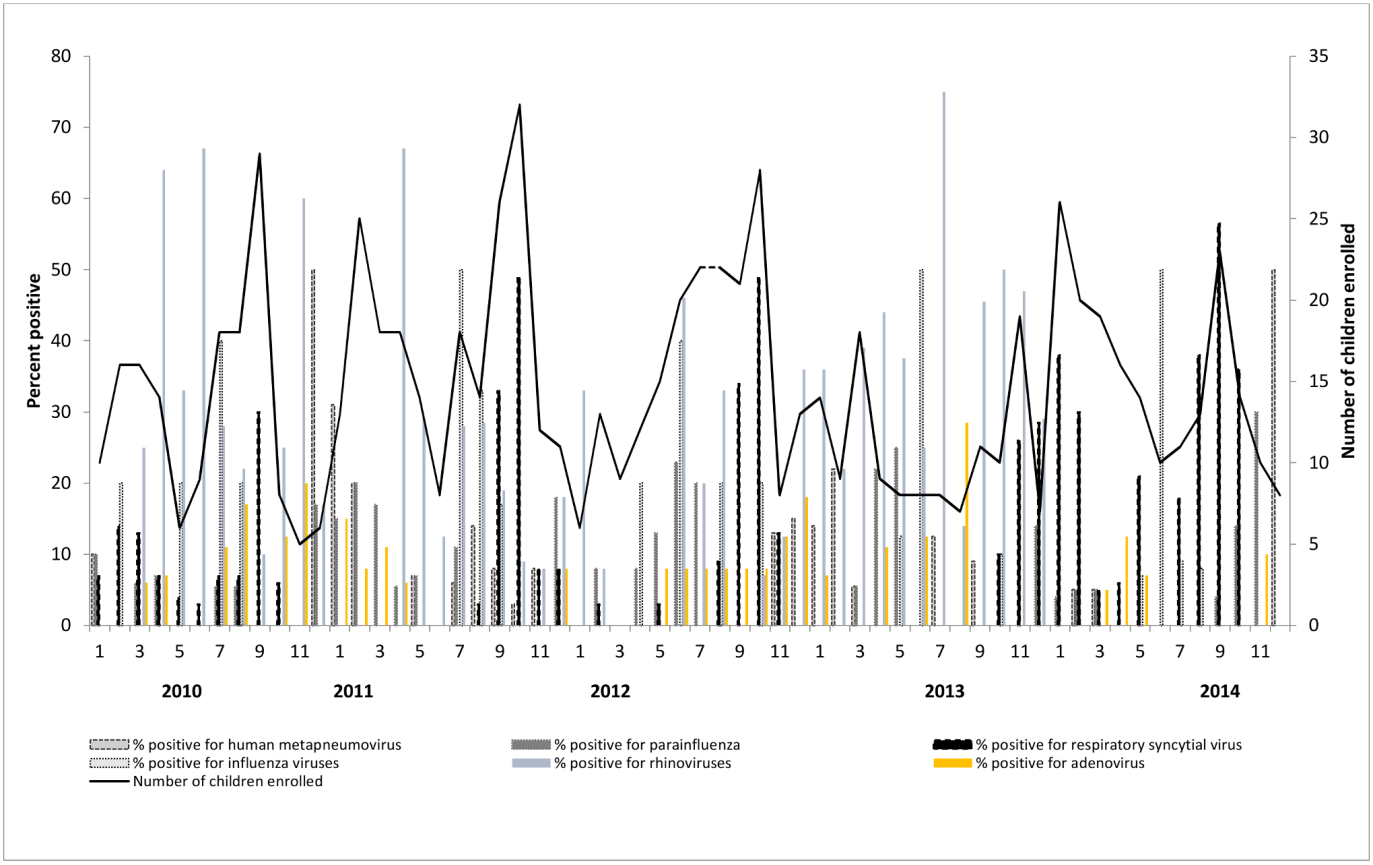
Monthly distribution of total number of enrolled hospitalized children with respiratory symptoms andproportion of children with respiratory symptoms positive for respiratory syncytial virus, human metapneumovirus, parainfluenza, influenza, adenovirus and rhinovirus infection in the four surveillance hospitals, Bangladesh, 2010–2014.

There were peaks in RSV-associated hospitalization during the last four months of the year in 2011 through 2014 when the temperature was cooler. Influenza activity peaked during June-July which is the rainy season in Bangladesh. There was no clear distinct seasonality for the other viruses (Fig 3).

### Health care utilization survey

During January 3-March 26, 2013 field researchers interviewed participants from 2,525 households and collected information on health care service utilization for 1,391 children aged <5 years. Among 1,391 children, 26 were hospitalized for respiratory symptoms (any two of the following: fever, cough or difficulty breathing) within the 12 months preceding the interview; 70% (19 out of 26) were male. Of the 26 children who were hospitalized with respiratory symptoms, 14 (54%) were hospitalized at the surveillance hospitals (four in Jahurul Islam Medical College Hospital, Kishoreganj, one in Shaheed Ziaur Rahman Medical College Hospital, Bogra, three in Comilla Medical College Hospital, Comilla and six in Sher-e-Bangla Medical College Hospital, Barisal).

### Annual incidence of hospitalized respiratory illness

We estimated that 2,356 children in 2010, 1,588 children in 2011, 1,193 children in 2012 973 in 2013 and 1,398 children in 2014 were hospitalized at the surveillance hospitals with respiratory illness. The estimated annual incidences per1000 children per year of all causes associated respiratory hospitalizations and respiratory viral infections associated hospitalizations were 19 and 10 in 2010, 12.5 and 4.5 in 2011, 9 and 2 in 2012, 7.4 and 1 in 2013 and 9 and 4 in 2014 (Table 2). The incidence over the four-year period of surveillance was highest for RSV associated respiratory hospitalization (Table 2).

**Table 2:** The incidences of respiratory viral pathogen specific hospitalized respiratory illness (per 1000 children per year) among children aged <5years in four surveillance hospitals, Bangladesh, 2010–2014.

Parameters	2010	2011	2012	2013	2014	2010–2014
Estimated number (95% CI)* **respiratory hospitalizations associated with**						
All cause	2356 (1986–2728)	1588 (1373–1804)	1193 (979–1347)	973 (804–1141)	1398 (0.3–0.6)	7508 (5328–8678)
Respiratory viruses	1277 (1034–1536)	570 (433–707)	297 (274–510)	137 (114–304)	570 (548–836)	2851 (2031–4351)
Respiratory syncytial virus	1049 (867–1232)	297 (198–403)	266 (175–365)	61 (15–122)	350 (236–471)	2023 (1461–2867)
Rhinovirus	608 (395–821)	281 (175–395)	160 (83–251)	388 (274–502)	NA	NA
Parainfluenza viruses 1–3	91 (15–213)	122 (53–205)	61 (15–122)	56 (8–99)	68 (15–129)	319(279–490)
Adenovirus	137 (30–274)	61 (15–122)	30 (0–76)	38 (0–91)	38 (0–91)	266 (177–355)
Human metapneumovirus	61 (0–152)	106 (38–190)	23 (0–68)	53 (8–115)	46 (8–99)	243 (194–389)
Influenza	76 (0–182)	46 (8–99)	23 (0–68)	76 (8–99)	68 (23–129)	289 (177–355)
**Proportion of children (95% CI)**¥**hospitalized at the sentinel hospitals in the preceding 12 months with respiratory symptoms**						
Four catchment areas	0.54 (0.3–0.7)	0.54 (0.3–0.7)	0.54 (0.3–0.7)	0.54 (0.3–0.7)	0.54 (0.3–0.7)	0.54 (0.3–0.7)
**Census population aged <5years**						
Four catchment areas	233428	236670	239912	243031	246190	239846
**Incidences/1000 children per year (95% CI)**						
All cause respiratory hospitalization	19 (18–25)	12.5 (12.0–16.5)	9 (8.8–12)	7.4 (7–10)	9 (9–12)	11.5 (10–12)
Respiratory virus-associated hospitalization	10 (9.6–14.0)	4.5 (4.0–6.5)	2 (2.5–5)	1 (1–3)	4 (2–6)	4 (2–6)
Respiratory syncytial virus-associated hospitalization	8.3 (8–11)	2.3 (2–4)	2 (1.5–3)	0.5 (0.1–1)	3 (2–4)	3 (2–3)
Rhinovirus-associated hospitalization	5 (3–7)	2 (1–3)	1 (0.6–2)	3 (2–4)	NA	NA
Parainfluenza virus-associated hospitalization	0.7 (0.1–2)	1.5 (0.5–2)	0.5 (0.1–1)	0.4 (0.1–1)	0.5 (0.1–1)	0.5 (0.4–0.8)
Adenovirus-associated hospitalization	1 (0.2–2)	1.5 (0.1–2)	0.5 (0–0.6)	0.3 (0–0.7)	0.3 (0–0.7)	0.4 (0.3–0.6)
Human metapneumo virus-associated hospitalization	0.5 (0–1)	0.8 (0.4–2)	0.2 (0–1)	0.4 (0.1–1)	0.3 (0.1–1)	0.4 (0.3–0.6)
Influenza-associated hospitalization	0.60 (0–2)	0.35 (0.1–1)	0.2 (0–1)	0.6 (0.1–1)	0.5 (0.1–1)	0.4 (0.3–6)

*The 95% CI around the estimates takes into account the variability in the percent positivity

^¥^ As the health utilization survey was a cross-sectional survey and was only conducted once the proportion of children who were hospitalized at the sentinel hospitals remains unchanged over the three years

## Discussion

Our data suggest that respiratory viruses, specifically RSV, are major contributors to childhood respiratory hospitalization in Bangladesh. Although our surveillance was only based in four hospitals, if we assume that our estimated incidences were similar across the country, then by multiplying the rates by the national population of 2,008,6376 of children aged <5 years we can estimate that approximately 80,345 (95% CI 79,790–80,902) children in 2014 were hospitalized with respiratory virus associated acute respiratory illness in Bangladesh. Given that only 35% of the children with ARI seek hospital care in Bangladesh [21], it is likely that the true burden of respiratory virus associated illness in children <5 years in Bangladesh could be higher. Such a high burden of respiratory virus associated severe illness and suboptimal health seeking behavior warrants the need for targeted interventions to lower the burden of viral respiratory infection in children <5 years.

The rates of RSV and influenza associated respiratory illness among hospitalized children aged <5 years were comparable to rates in other low and middle income countries including Kenya [24], Mozambique, South Africa [16], El Salvador [25], Costa Rica [17] and Thailand [15] which ranged between 3 to 10/1000 children per year for RSV and 0.3 to 2/1000 children per year for influenza. We did observe a high rate of RSV-associated hospitalizations in 2010. It is possible that 2010 was an exceptional year of increased RSV activity. Indeed a RSV-associated outbreak was reported from the country in the same year and population based data also suggest yearlong RSV activity in children aged<2 in 2010 [13, 26]. The RSV detection rate was lower in 2013 which could be due to the annual variation in the circulation pattern of RSV. RSV circulation varies from year to year and there can be large and small epidemics in some countries [23, 26]. The median age of the children we enrolled in 2013 was 7 months. It is possible that merely by chance we enrolled older children in 2013 which limited our capability to detect RSV positive children as the rate of RSV disease is highest in children aged <6 months [13, 27–30].

The RSV positivity rate in our study was higher among children aged <6 months. Different studies have noted the burden of hospitalization associated with RSV to be highest among children aged <6 months [13, 27–30]. The high rates of RSV-associated severe illness in very young children make RSV a suitable candidate for vaccine development. Currently, there are no effective antivirals or vaccines against RSV infection. Several RSV vaccines are being evaluated under clinical trials and have yet to be licensed [31]. Palivizumab, an expensive humanized monoclonal antibody [32] with proven efficacy in reducing hospitalization rates in high-risk infants and children [33], is only recommended for use in some middle and high-income countries [34, 35].

We detected a small number of children confirmed with influenza infection probably because we conducted the surveillance only four days a month. It is possible that we did not account for children with influenza who did not seek care at any hospital and who manifested differently from the surveillance system case-definition. Despite the small numbers of laboratory-confirmed influenza cases enrolled in this surveillance project, 35% (20 of 57) of all influenza positive children were aged <6 months. Unlike RSV, influenza is a vaccine preventable disease and maternal immunization against influenza protects children against influenza illness until they are eligible for vaccination at age ≥6 months [36–38]. Influenza vaccines are not routinely used in Bangladesh. Studies to evaluate the cost-effectiveness of immunization of pregnant women to prevent influenza among mothers and their infants aged <6 months may help public health officials explore the potential value of vaccinating pregnant women in Bangladesh.

More than 50% of the control children tested positive for a respiratory virus. Rhinovirus and adenovirus were the two most commonly identified viruses among control children. A study done in Alaska also frequently identified these two viruses among control children [39]. Our detection rate of 21% for rhinovirus and 12% for adenovirus were comparable to previous studies [40, 41]. Though rhinovirus was the second most common virus detected among hospitalized children after RSV, high detection rates among children without respiratory symptoms limit us from making inference about the causal relation between rhinovirus and illness. Respiratory viral detection rate of 1–2% for other viruses among asymptomatic hospitalized children was concordant with findings from other studies suggesting that probably the presence of these viruses are associated with respiratory symptoms. [39, 42].

Although there is limited information about the incidence of HMPV and PIV associated severe illness, like other published reports our data suggest that after RSV, PIVs were frequently identified among hospitalized children with respiratory illness [2]. The frequency of laboratory-confirmed infection with HMPV was 4% among our hospitalized children, a figure which was similar to those found in Hong Kong, Korea, USA and Netherlands [11–14]. The detection rate of HMPV, however, was 13% among hospitalized children with respiratory illness in neighboring India [43] and could be higher because of annual variation in viral activity. Despite differences in methodologies, the similarity in rates of hospitalized viral respiratory illness in diverse countries suggests that respiratory viruses indeed are important contributors of childhood hospitalization across the world.

As the surveillance was conducted only one day per week, the small number of sampled children limited our capacity to estimate precisely the total number of children that may have been hospitalized with respiratory illness. We did, however, account for the sampling fraction in our analyses and in addition the surveillance was conducted for five years which we believe contributed to generating credible estimates. We did not account for children who did not seek care at any hospital and who manifested differently from the surveillance systems’ case-definition. Our case definition included any two of the following symptoms: fever, cough and difficulty in breathing and it is likely that we missed children with ARI having high fever and runny nose. However a previous prospective population based study from our group showed that 100% of children with physician diagnosed pneumonia had cough and fever and >80% of the children had difficulty in breathing [13]. As the primary objective of the study was to estimate the burden of severe viral respiratory illness, inclusion of any two of the above mentioned symptoms in the case definition might have captured majority of the children with ALRI requiring admitted care on the sampling days. Our rates were comparable to a population-based study conducted in rural Bangladesh [44]. We considered the primary catchment areas of the hospitals to be areas where >60% of the patients resided which was an arbitrary cut-off. This cut off was chosen to balance the selection of sufficient cases from a large enough catchment area with the ability to precisely determine the proportion seeking care at the surveillance hospital versus another hospital. Choosing a very small proportion would have meant we had to canvas a very large catchment area to obtain a relatively small proportion of persons seeking care at the sentinel site and choosing a large proportion would have severely restricted the population at risk and the number of respiratory illness events; both extremes could have threatened the precision of our rate estimates. The health utilization survey was only done once in 2013, however we are not aware of any changes in health seeking behavior in Bangladesh and it is unlikely that proportion of children seeking care at the surveillance hospitals would have changed over time. Health care utilization is also limited by the capacity of the health system to provide care to the population. In Bangladesh there are 11 patients admitted for every 10 beds. [45]. We identified from the health utilization survey and surveillance that male children were hospitalized more frequently than female children which may have led to underestimation of the rates of severe respiratory illness among girls in the community. Although health seeking behavior in Bangladesh is skewed towards the male gender [46], male sex has also been identified as a risk factor for severe respiratory infection [44]. Also the surveillance hospitals were chosen from geographically diverse locations, our estimated rates were based on only four sites and the rate of respiratory viral infections among hospitalized children were likely different in other areas within Bangladesh; especially in the more densely populated urban areas of the country and thus our rates must be interpreted with caution. We used PCR technology to detect viruses which only confirms infection with a virus and does not confirm disease causation. We did not subtype the samples that were positive for rhinovirus. Indeed rhino virus subtype C has been associated with severe illness [47]. This limits our capacity to draw inference regarding the presence of rhinovirus and severity of respiratory illness in children. We also did not test the respiratory specimens for other viruses such as coronavirus, enterovirus or bocavirus which have been associated to respiratory illness. However due to logistical constraints, we were primarily interested in the viruses that were frequently associated with lower respiratory infections in children [48]. In addition we did not follow the asymptomatic children while they were hospitalized to see if they subsequently developed respiratory symptoms due to logistical constraints which may partly explain the high detection rate of rhinovirus and adenovirus among asymptomatic children. Lastly, we do not have the data on how many patients declined to participate, but the number of non-response was probably minimal. The children were already hospitalized and we only collected nasal and throat swabs from them through minimally invasive procedure so we believe unwillingness to participate in the surveillance might have been negligible.

Our data suggest that respiratory viruses caused a large number of hospitalizations among children aged <5 years in Bangladesh. Further development of effective vaccines against respiratory viral illness will play an instrumental role in lowering the burden of severe respiratory illness. In the meantime, research exploring the value of interventions such as frequent hand washing [49–51] may help in designing scalable cost-effective interventions in low-income settings like Bangladesh.
